# Exploring Paramedics’ Perspectives on Paediatric Head Injury in Prehospital Care: Qualitative Study

**DOI:** 10.1136/bmjpo-2025-004022

**Published:** 2025-12-24

**Authors:** Sara Alsuwais, Christopher Wibberley, Richard Body

**Affiliations:** 1 Department of Emergency Medical Services, King Saud bin Abdulaziz University for Health Sciences College of Applied Medical Sciences, Riyadh, Saudi Arabia; 2 King Abdullah International Medical Research Center, Riyadh, Saudi Arabia; 3 Division of Cardiovascular Sciences, Faculty of Biology, Medicine and Health, The University of Manchester, Manchester, UK; 4 Emergency Department, Manchester University NHS Foundation Trust, Manchester, UK

**Keywords:** Health services research, Child, Qualitative research

## Abstract

**Background:**

Paediatric head injury is a common reason for emergency calls. While most cases are mild, a small proportion deteriorates rapidly. Paramedics are often the first point of clinical contact, yet the absence of paediatric-specific tools, infrequent exposure and emotionally charged environments contributes to uncertainty. Paramedic perspectives in this context remain under-represented in the literature.

**Objective:**

To explore paramedics’ experiences, challenges and decision-making in the prehospital assessment of children with suspected head injuries and explore perceptions of existing hospital-based clinical decision rules and their potential use in out-of-hospital care.

**Methods:**

A qualitative study, guided by an interpretivist approach, was conducted with 37 paramedics from the North West Ambulance Service NHS Trust, United Kingdom. Purposive sampling captured a range of clinical grades and experience levels. Semistructured virtual interviews explored clinical assessment, decision-making, communication with families and views on current guidelines and clinical decision rules. The Paediatric Emergency Care Applied Research Network and the Children’s Head Injury Algorithm for the Prediction of Important Clinical Events were presented after participants described their usual practice. Interviews were audio-recorded, transcribed verbatim, anonymised and analysed inductively using reflexive thematic analysis.

**Results:**

Four inter-related themes captured the clinical, emotional and systemic realities of paediatric head injury assessment. Paramedics described the challenges in treating children, as developmental differences, limited communication and subtle or delayed symptoms required vigilance and adaptation. These were compounded by the paramedic’s own challenges, including low confidence from limited exposure, training gaps and the emotional and ethical pressures of safeguarding. Participants showed frustration over adult-oriented tools, rigid guidelines and remote decision-making that undermined autonomy. The role of clinical decision rules was seen positively for structure and defensibility, but with caution about safeguarding, compensatory physiology, contextual risk and their limited relevance to non-conveyance decisions in out-of-hospital care.

**Conclusions:**

Prehospital paediatric head injury assessment is shaped by intersecting clinical, emotional and systemic pressures. Improving care requires paediatric-specific decision tools, integrated training and system changes that support rather than professional judgement.

**WHAT IS ALREADY KNOWN ON THIS TOPIC**

- Most paediatric head injuries seen by paramedics are mild, but assessment is challenging.
- Paramedics report low confidence due to limited training and adult-focused tools.
- Hospital clinical decision rules (CDRs) (eg, Paediatric Emergency Care Applied Research Network, Children’s Head Injury Algorithm for the Prediction of Important Clinical Events) were not designed for prehospital care.

**WHAT THIS STUDY ADDS**

- Shows how emotional pressure, safeguarding and poor fit of current tools shape decisions.
- Paramedics welcome CDRs to support confidence and justify non-conveyance.
- Highlights caution about over-reliance on rules without clinical judgement.

**HOW THIS STUDY MIGHT AFFECT RESEARCH, PRACTICE OR POLICY**

- Calls for paediatric-specific CDRs adapted for out-of-hospital use.
- Underlines need for training that combines clinical, communication and safeguarding skills.
- Supports policies that strengthen safe, defensible non-conveyance decisions.

## Introduction

 Paediatric traumatic brain injury (TBI) is a leading cause of death and long-term disability in children worldwide.^1^ In the United Kingdom (UK), approximately 300 000 children attend emergency departments (EDs) each year with head injuries,^2^ the majority of which are mild. In an analysis of a UK-wide cohort of 5700 children, 79% presented with a Glasgow Coma Scale (GCS) score of 15 or were classified as ‘Alert’ he highest level of responsiveness on the AVPU (Alert, Voice, Pain, Unresponsive) scale.^3^ However, a small proportion is at risk of rapid deterioration, and early recognition and management remain critical to optimising outcomes.^4^

Regional data reflect this pattern, in the North West Children’s Major Trauma Network, 502 paediatric trauma admissions were recorded in 2023, with falls and non-accidental injury (NAI) as the most common mechanisms in infants under 1 year.^5^ Despite the clinical importance, there is limited UK-specific evidence describing the prehospital management of children with suspected TBI. Existing literature indicates that paramedics are often the first clinical contact, and their assessment and conveyance decisions have a significant impact on patient trajectories.^6 7^

The prehospital environment presents unique challenges. Assessment is frequently hindered by children’s compensatory physiology, which can mask severity, and by limited communication in very young or non-verbal patients. Scenes are often emotionally charged, with distressed caregivers and bystanders adding further pressure. Prior studies have shown a tendency towards overconveyance, with paramedics frequently transporting children with minor head injuries despite anticipating discharge without intervention.^8 9^ This is partly driven by a lack of structured, validated prehospital triage tools for paediatric head injury. In the ED, clinical decision rules (CDRs) have been shown to reduce unnecessary imaging and optimise resource use without compromising safety.^10^ Hospital-based rules such as Paediatric Emergency Care Applied Research Network (PECARN)^11^ and Children’s Head Injury Algorithm for the Prediction of Important Clinical Events (CHALICE),^12^ while widely validated,^13 14^ were developed to guide CT imaging decisions in the EDs and are not directly transferable to prehospital care, where the central question is whether to convey a child to hospital. Adapting such tools for prehospital use could support safe triage and reduce unwarranted conveyance, but no validated paediatric-specific prehospital rules currently exist.^8^ A recent systematic mapping review identified 25 hospital-based tools and highlighted clinical criteria such as skull fracture signs, scalp haematoma, GCS <15 and seizures that may be relevant for adaptation to prehospital practice.^15^

Despite their pivotal role, paramedics’ experiences, decision-making processes and views on guideline applicability for paediatric head injury remain under-represented in the literature. This study aimed to explore paramedics’ experiences, challenges and decision-making in the prehospital assessment of children with suspected head injuries, including their perspectives on the applicability of CDRs as potential triage tools to support non-conveyance decisions. By examining the operational, emotional and systemic factors influencing care, the findings seek to inform the development of decision support, training and system-level strategies to improve early management and patient outcomes.

## Methods

### Setting and participants

The study was conducted within the North West Ambulance Service NHS Trust (NWAS), UK, which serves over seven million people across urban and rural areas. NWAS is one of the largest UK ambulance trusts, covering a geographically and socioeconomically diverse population.^16^ Purposive sampling targeted registered NWAS paramedics with experience managing paediatric head injuries. Sample size was guided by the concept of information power,^17^ rather than the principle of data saturation, with the aim of achieving sufficient richness and diversity across clinical grade, years of experience and paediatric exposure to address the study objectives. Inclusion criteria were (1) current NWAS employment; (2) registration as a paramedic; (3) experience with paediatric prehospital care and (4) willingness to participate. Recruitment was coordinated through the NWAS Research and Development office, which circulated an invitation via the Trust’s internal staff email and intranet.

### Data collection

All interviews were conducted by the lead researcher (SA), a doctoral researcher and paramedic with experience in qualitative research and a focus on paediatric prehospital care. Interviews were conducted via Microsoft Teams. An interview guide, informed by literature and piloted with two external paramedics, explored clinical assessment, decision-making, communication with families and use of guidelines/tools. Examples of two CDRs (PECARN and CHALICE) were shown to prompt discussion on their relevance to out-of-hospital care, particularly in relation to decisions about conveyance and non-conveyance. These tools were selected as they are the most widely validated hospital-based rules for paediatric head injury and are frequently cited in UK and international guidelines.^1 11 13 14^ Other decision rules were considered less extensively validated in children and less directly relevant to the UK context and therefore were not included in this study. Interviews were audio-recorded, transcribed verbatim and anonymised.

### Data analysis

The study was conducted within a generic qualitative design situated in an interpretivist paradigm, which assumes that meaning is constructed through participants’ accounts and researcher interpretation.^18 19^ Data were analysed inductively using Braun and Clarke’s reflexive thematic analysis,^20^,^22^ following the Reflexive Thematic Analysis Reporting Guidelines.^23^ Codes were initially generated and grouped into candidate themes by the first author using ATLAS.ti Web version (ATLAS.ti Scientific Software Development GmbH, Berlin, Germany).^24^ These were then reviewed, refined and discussed with the supervisory team to enhance reflexivity, challenge interpretations and support analytic rigour. Reflexive notes documented analytic decisions and researcher influence throughout.

### Patient and public involvement

No patients or members of the public were directly involved in the design, conduct or analysis of this study, which focused on paramedics’ professional perspectives. Patient and parent perspectives are being explored in a separate study within the PhD Project.

## Results

### Participant characteristics

Recruitment was closed after the completion of 37 semistructured interviews, as this number provided sufficient information power and diversity across clinical grade, years of experience and paediatric exposure to meet the study aims. Participants had a mean age of 40 years (range 26–56) and an average of 15 years of clinical experience (range 6–33). Most were paramedics (n=20, 54.1%) or advanced paramedics (n=6, 16.2%), with the remainder holding other senior or specialist clinical roles, including Critical Care Paramedics, Clinical Leads and educators. All interviews were conducted virtually and lasted between 15 min and 59 min (mean 34 min), depending on participant availability and richness of responses. A full demographic summary is presented in table 1.

**Table 1:** Participant demographics (n=37)

Characteristic	Category	n
Age	26–35	11
	36–45	17
	46–56	9
Gender	Male	25
	Female	12
Highest level of education	BSc Paramedic Practice	16
	DipHE	10
	Foundation degree	2
	MSc	7
Clinical grade	Paramedic	20
	Advanced paramedic	6
	Critical care paramedic	4
	Senior paramedic team leader	2
	Sector clinical lead	2
	Advanced paramedic clinical lead	2
	Paramedic learning facilitator	1
Years of experience	6–10	14
	11–20	12
	21–33	11

### Overview of Themes

**Figure 1:**
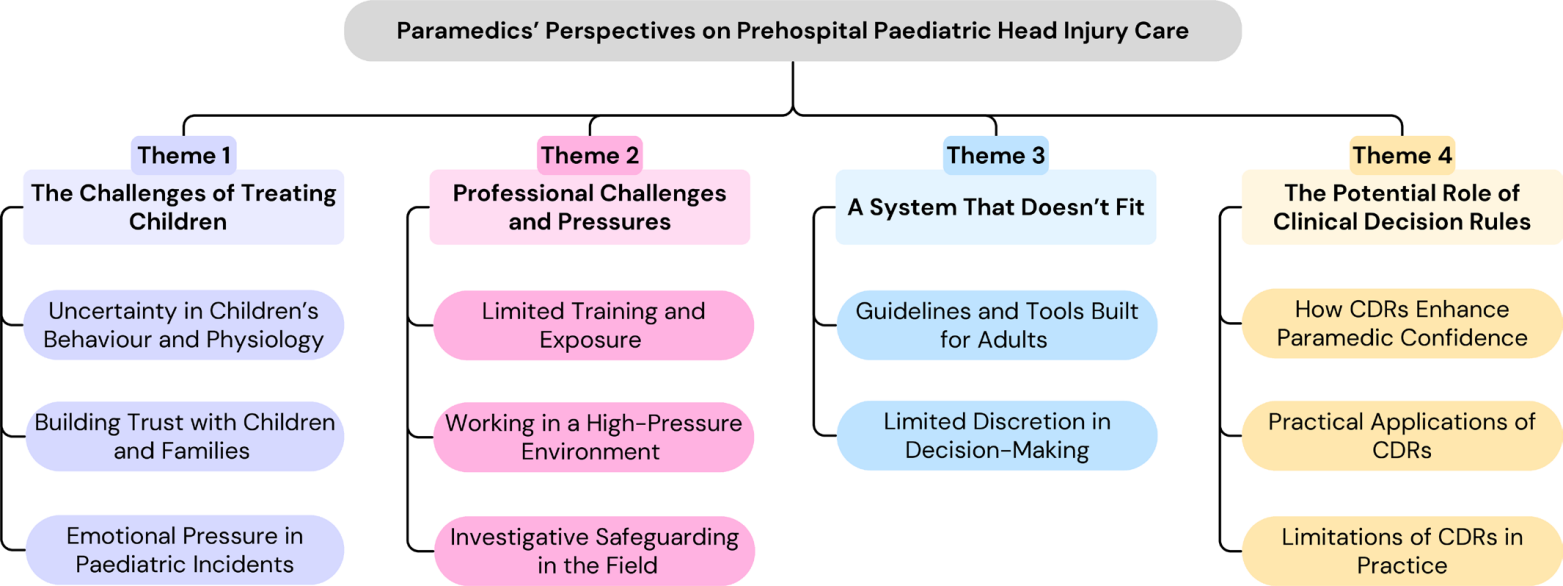
Thematic map of paramedics’ perspectives. CDRs, clinical decision rules.

Reflexive thematic analysis generated four interconnected themes: (1) the challenge of treating children, (2) professional challenges and pressures, (3) a system that doesn’t fit and (4) the potential role of clinical decision rules. Figure 1 presents the thematic map, illustrating how the themes and subthemes intersect. ‘The challenge of treating children’ captured the complexity of managing paediatric head injury, shaped by communication barriers, developmental differences, unpredictable physiology and the emotional presence of distressed parents. ‘Paramedic challenges in paediatric trauma care’ highlighted the lack of training and exposure to paediatric trauma, compounded by fear of hospital scrutiny and safeguarding responsibilities. ‘A system that doesn’t fit’ reflected the limitations of adult-oriented guidelines and restrictive protocols, which were seen to constrain professional discretion. ‘The potential role of clinical decision rules’ described how PECARN and CHALICE were valued for supporting assessment and non-conveyance, though participants emphasised they could not replace clinical judgement or address safeguarding concerns. Table 2 provides a concise summary of each theme with illustrative participant quotes.

**Table 2:** Summary of themes

Theme	Description	Example quote (participant)
The challenge of treating children	Managing paediatric head injuries was seen as highly complex due to communication barriers, developmental differences, unpredictable physiology and the influence of distressed parents. Building trust with children and families was essential but time-consuming.	‘They can compensate really well, and then suddenly… deteriorate very rapidly.’ (Paramedic 21) ‘But the biggest thing with children is they can't always explain how they feel. They don't understand that they feel dizzy, or that they've got a headache. Especially younger children’ (Paramedic 34)
Professional challenges and pressures	Limited training, low exposure to serious paediatric trauma and fear of judgement from hospital staff undermined confidence. Safeguarding concerns added emotional and professional pressure.	‘I think the biggest challenge… is looking at the child and thinking I don’t know what to do.’ (Paramedic 12)
A system that doesn’t fit	Guidelines and tools were often designed for adults, forcing paramedics to adapt them for children. Protocols could limit professional discretion, leading to perceived over-conveyance.	‘We don’t have many tools to assess them properly… it’s very much what we’ve learned for adults.’ (Paramedic 27)
The potential role of clinical decision rules	CDRs such as PECARN and CHALICE were viewed as useful for standardising assessment and supporting non-conveyance decisions but could not replace clinical judgement or address safeguarding risks.	‘Anything that aids decision-making for children would be a massive benefit.’ (Paramedic 36)

CDRs, clinical decision rules; CHALICE, Children’s Head Injury Algorithm for the Prediction of Important Clinical Events; PECARN, Paediatric Emergency Care Applied Research Network.

### Theme 1: The Challenge of Treating Children

Paramedics described paediatric head injury assessment as uniquely complex due to children’s limited ability to communicate, unpredictable clinical progress and emotional vulnerability. Children’s capacity to mask deterioration prompted a cautious approach, often leading to overtriage. ‘They can compensate really well… and then deteriorate very rapidly. So it’s always bearing that in the back of your mind’ (Paramedic 21).

This tendency to ‘mask’ injury meant paramedics often transported children even when signs appeared minor, choosing caution rather than risk a missed deterioration.

‘They can mask injuries particularly well. They can appear to be well… And so in response to that, we might compensate for that with over-triage… we might be overcautious with this group of patients’ (Paramedic 6).

Communication barriers further complicated assessment. Younger children often lacked the vocabulary to describe symptoms, while some minimised them to avoid hospital transfer. This uncertainty reinforced a defensive style of practice, where the absence of clear symptoms was not always reassuring.

‘But the biggest thing with children is they can't always explain how they feel. They don't understand that they feel dizzy, or that they've got a headache. Especially younger children, and sometimes even older ones just don't want to tell you what’s going on, or they play it down because they don’t want to go to hospital’ (Paramedic 34)

Building trust with both child and parent was seen as essential to cooperation, using techniques such as distraction, involving parents and delaying examination until rapport was established. ‘Children pick up on their parents’ emotions… a lot of it is about trying to calm the parents down… so you can perform a better assessment’ (Paramedic 32).

Paediatric incidents also carried a higher emotional load, especially for parents themselves. Scenes could become chaotic, increasing stress and sometimes prompting a ‘scoop and run’ approach to get the child into hospital quickly. The interaction between emotional intensity and clinical judgement was clear; emotional strain overrode the measured, stepwise assessment used with adults. ‘It’s a challenging job, the outcome is, ‘I want to get this away from me as quickly as possible and give it to someone else’ (Paramedic 23)

### Theme 2: Professional Challenges and Pressures

Limited training and infrequent exposure to paediatric trauma left many feeling underprepared and lacking confidence. Undergraduate and Continuing Professional Development provision was seen as heavily adult-focused, with rare opportunities for realistic paediatric simulation. ‘We focus a lot of our training on adult injuries… enhanced training would definitely benefit the majority of paramedics’ (Paramedic 33).

Some reflected that this gap left them professionally vulnerable in real incidents, where high-stakes decisions had to be made under pressure.

‘I think the biggest challenge personally in my practice… looking at the child and thinking I don't know what to do, I always used to worry about that thinking I'm just going to be frozen or I'm not going to know how to move on and how to act’ (Paramedic 12).

This self-doubt was compounded by perceived scrutiny from hospital-based paediatric staff during handover, reinforcing a culture of caution and leading to unnecessary conveyance in some cases. There is very much a blame culture… sometimes people will just take them to hospital when they may not necessarily need to go’. (Paramedic 26)

Safeguarding responsibilities, particularly in suspected NAI, created additional stress. Participants valued their unique ability to assess the child’s home environment but feared missing subtle signs, often erring on the side of hospital referral. ‘We’re in the best position to make an educated judgement about someone’s living situation… hospital clinicians don’t get to see that’ (Paramedic 24).

### Theme 3: A System That Doesn’t Fit

Participants consistently described out-of-hospital guidelines and assessment tools as adult-oriented and poorly adapted to children’s developmental differences, forcing them to adapt or improvise in paediatric cases. Tools such as the GCS were seen as less reliable in young children due to developmental and communication differences. ‘GCS is inconsistently applied… even more so with a child because a lot of it is more subjective’. (Paramedic 23)

Some participants went further, questioning how accurately GCS is scored in real-world paediatric cases and suggesting ways this could be tested.

‘How accurately people do score GCS in paediatrics. You could use a video of a child and just get everyone to score the GCS on the child. Because, again, paediatric GCS isn’t something we do very often, especially in a trauma scenario. I think there’s a definite skill element to scoring GCS, it’s something we do so regularly with adults’ (Paramedic 25)

They reported feeling less confident when applying it to children, particularly those under 5. Developmental differences and limited verbal abilities were seen to reduce its reliability and complicate interpretation. As one paramedic explained:

‘With adults, I feel more confident in the application. But when it comes to paediatric GCS, the difference between calculating it for a 2-year-old as opposed to a 10- or a 15-year-old presents its own challenges’ (Paramedic 21).

In addition, protocols limiting non-conveyance were viewed as undermining autonomy and professional judgement, even when the injury appeared minor. ‘I’m an autonomous clinician… this idea that we’re not trusted enough to make a decision unless a little flowchart tells us it’s correct is ridiculous’ (Paramedic 32).

Several participants described cases where children who appeared well were nonetheless conveyed to hospital because the guidelines left no flexibility. While intended to minimise risk, many felt these restrictions encouraged overconveyance, reinforced a culture of risk aversion and weakened professional judgement through rigid flowcharts.

### Theme 4: The Potential Role of CDRs

CDRs such as PECARN and CHALICE were generally welcomed as structured, evidence-based aids that could align prehospital and hospital decision-making, boost confidence and support communication with parents. ‘Anything that aids decision-making for children would be a massive benefit… boosting confidence will lead to better patient outcomes’ (Paramedic 36)

Many paramedics indicated they would feel comfortable using a well-designed CDR in the assessment and management of paediatric head injuries. This confidence stemmed from several key factors. They valued the fact that many CDRs are already used within hospital settings and have been subjected to clinical validation. Knowing that these tools are trusted by ED staff, offered reassurance that using them in the out-of-hospital environment would be both acceptable and professionally supported.

‘We’re going to use the same assessments that you're getting in hospital, because that’s how you make the best decisions. I can't see anything in either of (PECARN/CHALICE) that I could not do in someone’s house or on the side of a road… They're very succinct and very good ways of working out how bad a head injury in a child is’ (Paramedic 32)

CDRs were seen as helpful in justifying non-conveyance decisions, determining the most appropriate destination and standardising care. Some described them as ‘triage tool’ offering defensibility when deciding to discharge with advice or refer into community services.

‘If I’ve worked through this flowchart and I’ve got a good feeling that the child is well in themselves, with no severe features, I might decide they can be discharged with advice. It’s almost like, at the point where no CT is required, there should be a question of whether we need to convey this patient to hospital… It’s almost a triage tool’ (Paramedic 12).

‘It would certainly guide the clinician on where to take that patient. So, if the child met the criteria and was going to require a CT scan, then you'd probably take them to the most appropriate centre, which could be a trauma centre or trauma unit, rather than a local hospital’ (Paramedic 8).

Participants saw CDRs as a way to strengthen communication with parents in emotionally charged situations, by making decision-making more transparent.

‘If we’re applying exactly the same validated tool in the prehospital setting as in the hospital, and parents know that, then maybe it adds more weight to the clinical assessment we’ve done… it’d be a good tool to justify why a child wouldn’t need to go to hospital’ (Paramedic 29)

However, participants cautioned against over-reliance, noting that CDRs cannot replace safeguarding judgement, detect delayed deterioration or overcome communication barriers. ‘Unless I’m 100% sure, I wouldn’t leave a child at home… anything can change within an hour’ (Paramedic 26).

‘It’s not just about the child’s injuries… there are social factors as well’ (Paramedic 28).

The consensus was that CDRs should complement, not replace, professional judgement and that their greatest potential value in the out-of-hospital setting lies in supporting, but not determining, non-conveyance decisions, enhancing defensibility and improving communication, while safeguarding and contextual factors remain the responsibility of the clinician.

## Discussion

This study explored paramedics’ experiences in the prehospital assessment of paediatric head injuries, identifying four themes: the challenges of treating children, the personal and professional challenges faced by paramedics, the mismatch between existing systems and paediatric needs and the potential but limited role of CDRs. The findings show how developmental differences, communication barriers, emotional intensity and systemic constraints converge to influence decision-making. They revealed paediatric head injury management as a high-stakes, low-frequency task requiring not only clinical expertise but also emotional regulation, safeguarding awareness and adaptive reasoning under pressure.

The findings align with earlier studies describing paediatric calls as infrequent but high-risk events, with children’s compensatory physiology, limited communication skills and variable behaviour creating assessment uncertainty.^25^,^28^ Like previous research, participants reported reduced confidence due to limited paediatric training and exposure,^29^ and frustration with adult-oriented tools such as the GCS when applied to young or non-verbal children.^30 31^ However, this study extends existing literature by linking these challenges to perceived inspection from hospital teams,^32^ the emotional weight of safeguarding decisions^33^ and the operational pressures of managing chaotic or emotionally charged scenes.^9 27 28 34^ It also adds nuance to the discussion of CDRs, showing that while paramedics value their structure and alignment with hospital practice,^11 35^ they remain cautious about over-reliance, particularly in safeguarding and subtle deterioration scenarios.^9 36 37^ This aligns with recent work,^15^ which identified hospital-based CDR elements that may be suitable for adaptation to prehospital use, while emphasising the need for contextual factors to be considered in out-of-hospital decision-making.

Improving paediatric out-of-hospital care requires more than introducing new assessment tools; it demands integrated strategies that combine adapted paediatric-specific CDRs with enhanced training in clinical assessment, communication, parental engagement, safeguarding and stress management. System-level changes should ensure that guidelines support rather than restrict professional judgement, and that interprofessional training builds mutual trust between prehospital and hospital teams. Policymakers should recognise that non-conveyance decisions in paediatric head injury are influenced as much by emotional, environmental and safeguarding considerations as by clinical findings.

Future research should assess the real-world application of paediatric-adapted CDRs in prehospital settings, including their effect on decision-making, conveyance rates and patient outcomes. Comparative studies across ambulance services could clarify how organisational structures and resources influence paediatric trauma management. Further work should explore how targeted training interventions, particularly simulation-based and interprofessional approaches, affect paramedic confidence, safeguarding detection and family communication in paediatric emergencies.

### Strengths and limitations

A key strength of this study is its qualitative depth, drawing on accounts from paramedics with varied clinical backgrounds and years of service. The reflexive thematic approach enabled exploration of both clinical and interpersonal dynamics often absent in quantitative research. This study contributes to the limited qualitative literature exploring the operational and emotional dimensions of paramedic care in paediatric head injury emergencies. While previous literature has identified training gaps and tool limitations in paediatric care, this work adds depth to our understanding of professional uncertainty, confidence and system-level tensions as experienced by frontline clinicians. It further offers a practical perspective on the potential integration of CDRs in prehospital settings, highlighting considerations that extend beyond clinical utility alone.

Several limitations should be acknowledged. The study was conducted within a single NHS ambulance service (NWAS), which may reflect local organisational policies and pathways. Although NWAS is one of the largest UK ambulance trusts, serving a socioeconomically and geographically diverse population, transferability to other services may be limited. Recruitment may have reflected the perspectives of paramedics more confident or motivated to discuss paediatric care. Patients and the public were not directly involved in this study, which is a limitation in terms of incorporating family perspectives. However, their views are being examined in a separate study within the wider programme of work. Virtual interviews enabled participation across geographic areas but may have influenced rapport and reduced visibility of non-verbal cues. Finally, PECARN and CHALICE were introduced only after participants had described the tools and approaches they currently used. This sequencing helped minimise bias and ensured that discussion of these CDRs built on, rather than replaced, participants’ own perspectives. While their inclusion still directed some focus towards formalised decision aids, it enabled richer exploration of how such tools might be integrated into out-of-hospital practice.

## Conclusion

Paramedic assessment of paediatric head injuries in the prehospital setting is shaped by clinical uncertainty, emotional pressures and systems designed primarily for adults. This study highlights the need for decision-support tools, such as paediatric-adapted CDRs, that strengthen rather than replace clinical judgement, alongside targeted training in communication, safeguarding and stress management. Policies and guidelines must reflect the realities of frontline practice, enabling flexible decision-making that integrates developmental, social and contextual factors. Improving these areas offers the potential to enhance confidence, reduce unnecessary conveyance and safeguard children more effectively.

## Data Availability

Data are available upon reasonable request.
