# Supplementary material for: Exploring Paramedics’ Perspectives on Paediatric Head Injury in Prehospital Care: Qualitative Study
Source: BMJ Paediatr Open. 2025 Dec 24;9(1):e004022. doi: 10.1136/bmjpo-2025-004022 (PMC12742124; doi:10.1136/bmjpo-2025-004022)
Supplement: Supplementary file 1 [file bmjpo-2025-004022.reviewer_comments.pdf]

PEER REVIEW HISTORY

BMJ Paediatrics Open publishes all reviews undertaken for accepted manuscripts. Reviewers are asked to complete a checklist review form and are provided with free text boxes to elaborate on their assessment. These free text comments are reproduced below.

ARTICLE DETAILS

**TITLE (PROVISIONAL)**	Exploring Paramedics' Perspectives on Paediatric Head Injury in Prehospital Care: Qualitative Study
**AUTHORS**	Alsuwais, Sara; Wibberley, Christopher; Body, Richard

VERSION 1 - REVIEW

**REVIEWER NAME**	Alyesha Proctor
**REVIEWER AFFILIATION**	University of the West of England
**REVIEWER CONFLICT OF INTEREST**	N/A
**AI DISCLOSURE**	No
**DATE REVIEW RETURNED**	15-Sep-2025

**GENERAL COMMENTS**	Thank you for the opportunity to review this manuscript exploring paramedics' perspectives on paediatric head injury in pre-hospital care. This is an area that I am particularly interested in and therefore I thoroughly enjoyed reading it, and could resonate with a lot of the findings.<br><br>I have a few minor suggestions:<br><br>- Why did you only include NWAS paramedics? Why could this study not recruit paramedics from across the UK using social media and professional connections?<br><br>- The methods section is limited and left me with lots of questions. What is the theoretical framework? Generic qualitative research? It certainly has not engaged deeply with phenomenological assumptions. What do you mean by 'recruited by internal communications'? More detail is needed about recruitment. How was consent obtained? Who conducted the interviews? How many people were recruited before you had the sample of 37? You mentioned information power but did not really tell the reader why that meant the sample is 37.<br><br>- Why have you not involved patients and the public with this work?<br><br>- I think more illustrative quotes would be helpful and you could make the names of the themes more 'fun' and relatable.<br><br>- A limitations subheading within the discussion would be helpful.<br><br>- Consider using and including this reference which I could not see in your reference list: Proctor, A., Lyttle, M., Voss, S., Shaw, P., Simpson, J. and Benger, J. Which elements of hospital-based clinical decision support tools for the assessment and management of children with head injury can be adapted for use by paramedics in

	prehospital care? A systematic mapping review and narrative synthesis<br><br>- It might be worth telling the reader why you selected PECARN and CHALICE (most validated hospital-based tools for children with head injury). Because there are obviously others such as CATCH and NEXUS2.<br><br>-I think it should be made clearer that PECARN and CHALICE are not wholly suitable to pre-hospital care and cannot directly be implemented in this setting as they are, since they aim to determine whether a CT scan is required, rather than conveyance out-of-hospital.<br><br>Thank you again for the opportunity to review this article!

**REVIEWER NAME**	Sarah Voss
**REVIEWER AFFILIATION**	None disclosed
**REVIEWER CONFLICT OF INTEREST**	N/A
**AI DISCLOSURE**	No
**DATE REVIEW RETURNED**	24-Sep-2025

**GENERAL COMMENTS**	This paper describes a qualitative interview study with paramedics regarding conveyance decision making for children with minor head injury. It is a well-written report and I believe it will be of interest to the readership.<br>The introduction is clear and relevant and the findings are relevant, well described and supported by evidence. My main suggestions for improvement are focussed on the reporting of the methods and the analysis.<br>1. In the abstract and methods the authors report that they conducted an interpretivist framework analysis and that results were analysed inductively using reflexive thematic analysis (RTA). This is unclear and comes across as slightly contradictory. I can understand how RTA was applied to the data to code it and develop themes but it is not clear if/how an interpretivist framework was developed and then used to do a further round of deductive coding. This needs to be explained or amended.<br>2. It is not clear how paramedics were recruited and selected. The authors state that 'recruitment was via internal communication channels'. More detail is needed on how the study was publicised, was this across the whole Trust? How did participants then get involved? How was eligibility confirmed? How were they recruited and consented? Verbal or written consent? How were those how expressed an interest sampled? It says that purposive sampling was adopted. If so, how many paramedics applied to take part and how many were eventually selected? Was a sampling frame used? If all eligible paramedics who volunteered were recruited then it is likely to be a convenience sample.<br>Minor points:<br>I suggest avoiding abbreviations in the abstract<br>The term out-of-hospital may be preferable to prehospital. This is because prehospital infers that admission to hospital is part of the patient trajectory but this research is relevant to opportunities to avoid conveyance/admission<br>Details on who collected and analysed data should be reported

	The length of time the interviews lasted should be reported in the results rather than methods and it would be helpful to have additional detail on this (min/max/mean duration)

VERSION 1 – AUTHOR RESPONSE

Reviewer & Comment

**Reviewer 1 – Dr. Alyesha Proctor**		
1.	Why did you only include NWAS paramedics? Why could this study not recruit paramedics from across the UK using social media and professional connections?	This study was embedded within the NWAS, where research governance and ethical approvals were already in place. Expanding recruitment across multiple NHS ambulance trusts or via social media would have raised challenges in verifying eligibility, which was beyond the scope of this project. We have acknowledged the single-site design as a limitation.
2.	<u>The methods section is limited and left me with lots of questions.</u>	Thank you for raising these points.<br>We have clarified each area in the Methods section as follows:
a.	What is the theoretical framework? Generic qualitative research? It certainly has not engaged deeply with phenomenological assumptions.	the study employed a generic qualitative design supported by an interpretivist approach, which is appropriate for exploring experiences and meanings in context without adhering to the procedural constraints of specific traditions such as phenomenology. This has been added to the Methods.
b.	What do you mean by 'recruited by internal communications'?<br>More detail is needed about recruitment.	Recruitment was coordinated through the NWAS Research & Development office, which circulated an invitation via internal staff email and intranet. Updated in Methods.
c.	How was consent obtained?	We have clarified in the Ethics section that written informed consent was obtained electronically from all participants before the interviews
d.	Who conducted the interviews?	We added all interviews were conducted by the lead researcher (SA).

e.	How many people were recruited before you had the sample of 37?	37 paramedics responded to the invitation, and all were eligible and interviewed. Clarified in Methods.
f.	You mentioned information power but did not really tell the reader why that meant the sample is 37.	We have expanded this, linking information power to sample adequacy.
3.	Why have you not involved patients and the public with this work?	This study focused on paramedics' professional perspectives; therefore, patients/public were not directly involved. However, patient/parent perspectives are being explored in a separate study within the wider programme. The absence of PPI is acknowledged as a limitation.
4.	I think more illustrative quotes would be helpful and you could make the names of the themes more 'fun' and relatable.	We have added additional illustrative quotes in the Results section to further demonstrate how themes are grounded in participant experiences. We have retained the original theme names, as we believe they best reflect the analytic focus and maintain clarity for a clinical and academic readership.
5.	A limitations subheading within the discussion would be helpful.	We have added a "Strengths and limitations" subsection in the Discussion.
6.	Consider using and including this reference which I could not see in your reference list: Proctor, A., Lyttle, M., Voss, S., Shaw, P., Simpson, J. and Benger, J. Which elements of hospital-based clinical decision support tools for the assessment and management of children with head injury can be adapted for use by paramedics in prehospital care? A systematic mapping review and narrative synthesis	This reference has been incorporated into both the Introduction and Discussion.
7.	It might be worth telling the reader why you selected PECARN and CHALICE (most validated hospital-based tools for children with head injury). Because there are obviously others such as CATCH and NEXUS2.	This is now clarified in the Data Collection section.

8.	I think it should be made clearer that PECARN and CHALICE are not wholly suitable to pre-hospital care and cannot directly be implemented in this setting as they are, since they aim to determine whether a CT scan is required, rather than conveyance out-of-hospital.	We have clarified this in the Introduction.
**Reviewer 2 – Prof. Sarah Voss**		
1.	In the abstract and methods the authors report that they conducted an interpretivist framework analysis and that results were analysed inductively using reflexive thematic analysis (RTA). This is unclear and comes across as slightly contradictory. I can understand how RTA was applied to the data to code it and develop themes but it is not clear if/how an interpretivist framework was developed and then used to do a further round of deductive coding. This needs to be explained or amended.	The study was conducted under an interpretivist approach, with data analysed inductively using Braun & Clarke's reflexive thematic analysis. We did not use framework analysis or deductive coding. We have amended the Abstract and Methods to avoid ambiguity.
2.	It is not clear how paramedics were recruited and selected. The authors state that 'recruitment was via internal communication channels'. More detail is needed on how the study was publicised, was this across the whole Trust? How did participants then get involved? How was eligibility confirmed? How were they recruited and consented? Verbal or written consent? How were those how expressed an interest sampled? It says that purposive sampling was adopted. If so, how many paramedics applied to take part and how many were eventually selected? Was a sampling frame used? If all eligible paramedics who volunteered were recruited then it is likely to be a convenience sample.	Recruitment was coordinated via NWAS R&D office, circulated to all staff by internal email/intranet. Interested paramedics contacted the lead researcher, eligibility was confirmed against pre-specified criteria, and written informed consent was obtained electronically. A purposive sampling approach was applied to capture variation in grade, experience, and paediatric exposure. Recruitment closed at 37 eligible participants, achieving sufficient information power. Methods updated accordingly.
3.	**<u>Minor Points</u>**	
a.	I suggest avoiding abbreviations in the abstract	The Abstract has been revised to avoid abbreviations.
b.	The term out-of-hospital may be preferable to prehospital. This is because prehospital	We used both "prehospital" and "out-of-hospital" depending on context.

	infers that admission to hospital is part of the patient trajectory but this research is relevant to opportunities to avoid conveyance/admission	“Prehospital” is retained where it reflects established usage in the emergency care literature, while “out-of-hospital” is used when discussing conveyance and non-conveyance decisions.
c.	Details on who collected and analysed data should be reported	We have clarified in the Methods that all interviews were conducted by the first author, a doctoral researcher and registered paramedic with qualitative research experience. We have also specified that data analysis was initially conducted by the first author using reflexive thematic analysis, with regular discussion and refinement in the supervisory team to enhance reflexivity and analytic rigour
d.	The length of time the interviews lasted should be reported in the results rather than methods and it would be helpful to have additional detail on this (min/max/mean duration)	We have moved the interview duration from the Methods to the Results section and added further details.

VERSION 2 - REVIEW

**REVIEWER NAME**	Alyesha Proctor
**REVIEWER AFFILIATION**	University of the West of England
**REVIEWER CONFLICT OF INTEREST**	N/A
**AI DISCLOSURE**	No
**DATE REVIEW RETURNED**	24-Nov-2025

**GENERAL COMMENTS**	Thank you for your responses to the comments. I am happy that all of my comments have been answered satisfactorily. It is a very interesting article and I thoroughly enjoyed reading and reviewing it.
